# Effect of GNWs/NiO-WO_3_/GNWs Heterostructure for NO_2_ Gas Sensing at Room Temperature

**DOI:** 10.3390/s22020626

**Published:** 2022-01-14

**Authors:** Seokhun Kwon, Seokwon Lee, Joouk Kim, Chulmin Park, Hosung Jung, Hyungchul Kim, Chulsoo Kim, Hyunil Kang

**Affiliations:** 1 Department of Electrical Engineering, Hanbat National University, Dongseo-daero, Yuseong-gu, Daejeon 34158, Korea; kwon1567@naver.com (S.K.); dltjrdnjs000@naver.com (S.L.); msdkcs1@gmail.com (C.K.); 2 Korea Railroad Research Insititute, 176 Cheoldobangmulgwan-ro, Uiwang-si 16105, Korea; jookim@krri.re.kr (J.K.); cmpark@krri.re.kr (C.P.); hsjung@krri.re.kr (H.J.); hckim@krri.re.kr (H.K.)

**Keywords:** gas sensor, GNWs, heterostructure, NO_2_, room temperature

## Abstract

Recently, as air pollution and particulate matter worsen, the importance of a platform that can monitor the air environment is emerging. Especially, among air pollutants, nitrogen dioxide (NO_2_) is a toxic gas that can not only generate secondary particulate matter, but can also derive numerous toxic gases. To detect such NO_2_ gas at low concentration, we fabricated a GNWs/NiO-WO_3_/GNWs heterostructure-based gas sensor using microwave plasma-enhanced chemical vapor deposition (MPECVD) and sputter, and we confirmed the NO_2_ detection characteristics between 10 and 50 ppm at room temperature. The morphology and carbon lattice characteristics of the sensing layer were investigated using field emission scanning electron microscopy (FESEM) and Raman spectroscopy. In the gas detection measurement, the resistance negative change according to the NO_2_ gas concentration was recorded. Moreover, it reacted even at low concentrations such as 5–7 ppm, and showed excellent recovery characteristics of more than 98%. Furthermore, it also showed a change in which the reactivity decreased with respect to humidity of 33% and 66%.

## 1. Introduction

In recent years, the quality of human activities has been greatly improved due to the development of civilization with advanced industry such as bio-health care, electric automobiles, IoT (Internet of Things), artificial intelligence (AI), batteries, and semiconductors. However, there are global issues of duplicity for industrial development, such as the destruction of natural ecosystems, depletion of fossil fuels, and lack of resources [1,2,3]. The most typical problems are air pollution and particulate matters, which cause serious environmental pollution. These two problems are very dangerous factors that can directly damage human breathing and skin tissue, and we are always exposed to them [4,5,6]. To minimize damage and alleviate the problems, eco-friendly policies to reduce the causes of environmental pollution are being implemented, but the results are still not satisfactory. Although it is not possible to create a clean air environment in a short time, continuous monitoring and analysis of air environment will be a necessary foundation. As a representative environmental pollutant gas with one of the toxic gases, nitrogen dioxide (NO_2_) is pointed out to be a source of air pollution, which occurs during the combustion process while burning fuel at a high temperature [7,8,9]. In addition, NO_2_ can derive nitric acid (HNO_3_) by reacting with ozone (O_3_) in the air, and then it forms ammonium nitrate (NH_4_NO_3_) under subsidiary reaction with ammonia (NH_3_) in the air, causing secondary particulate matter [10,11]. Thus, detection of NO_2_ is highly significant in the monitoring and analysis of air environments.

The scientific techniques for detection of diverse harmful gas including NO_2_ have been reported using metal–oxide semiconductors (MOS), graphene-centered carbon materials, polymers, and transition metal dichalcogenide (TMD) as sensing layers [12,13,14,15]. Among them, although an MOS-based gas-sensor platform offers low cost, solid-state, and simplicity of operation, it must be considered a micro heating system for operating temperature in its design and platform structure [16]. In addition, the conductive polymers include polypyrrole (PPy), polythiophere (PTh), and polyaniline (PANI), and are widely used owing to their redox properties, surface-charge-tunable properties and good flexibility, but there still remain some drawbacks, such as their low sensitivity, low selectivity, and high detection limit [17]. In TMD materials, the most popular and attractive materials for sensing material are molybdenum disulfide (MoS_2_) and tungsten disulfide (WS_2_), which provide low electrical noise, low detection limit, tunable band gap and high mobility, while they suffer from low selectivity and sensitivity as disadvantages [18,19,20]. After the discovery of graphene, carbon materials showed desirable utility in accordance with gas-sensor applications. Particularly, the graphene series (graphene, graphene oxide (GO), including reduced graphene oxide (rGO)) are remarkable sensing materials due to their wide surface area, high carrier mobility and concentration, leading to good gas-sensing performance upon exposure to NO_2_ [21,22,23]. Nevertheless, required by the necessity of detection ability for low concentration (ppb-ppm level) and the significance of porosity, a three-dimensional nanostructure leading to the expansion of active sites by surface modification or one-step fabrication became crucial.

In this study, we used graphene nanowalls (GNWs) while keeping the advantages of graphene via microwave plasma-enhanced chemical vapor deposition (MPECVD). GNWs are three-dimensional materials with high porosity, which enable molecules of target gases to induce more absorption onto its surface and active sites [24,25]. Although we found that the pure GNWs enabled us to detect the target gases from our previous study, it required drastic enhancement of gas-sensing performance, and hence, we introduced a NiO-WO_3_ layer on the surface of GNWs, where NiO-WO_3_ can serve as a metallic catalyst that elevates detection signal by NO_2_ gas molecules [26,27,28]. Typically, single-catalyst materials have been used from former studies, while we chose hybrid-catalyst materials by the co-sputtering system. Then, secondary GNWs were formed on the NiO-WO_3_/GNWs structure for securing a larger adsorption area. Consequently, measurement of NO_2_ with several concentrations was achieved by the GNWs/NiO-WO_3_/GNWs heterostructure in this study.

## 2. Materials and Methods

### 2.1. Preparation of the GNWs/NiO-WO_3_/GNWs Heterostructure

**Figure 1:**
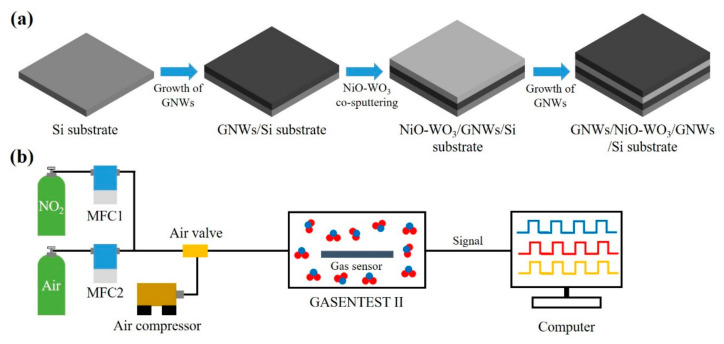
Schematic illustration of GNWs/NiO-WO_3_/GNWs heterostructure-based NO_2_ gas sensor. (**a**) Fabrication process of GNWs/NiO-WO_3_/GNWs heterostructure-based NO_2_ gas sensor using PECVD and sputtering system. (**b**) Measurement of sensing performance under exposure to NO_2_ gas.

For preparation of the GNWs/NiO-WO_3_/GNWs heterostructure, as shown in Figure 1a, first, GNWs were grown on Si substrate using microwave plasma enhanced chemical vapor deposition (Woosin CryoVac, Uiwang, Korea; CVD-R2) under conditions such as microwave power (1200 W), reaction gas ratio (H_2_:CH_4_ = 40:20 sccm), substrate temperature (600 °C), synthesis time (15 min), and working pressure (25 mTorr). In the formation of GNWs, the state of radicals affects the growth steps of GNWs, such as carbon nucleation, graphene domain, and growth rate. For these, two CVD reactions should be kept and accompanied: (i) CH, CH_2_, and CH_3_ radicals based on high energy and unstable state should stay until arrival onto the substrate under the appropriate microwave power, reaction gas ratio, synthesis time, and working pressure; and (ii) the radicals that have arrived on substrate should join the chemical reaction with dehydration under the proper temperature. Hence the chosen MPECVD condition is optimal for successful GNWs growth. Afterwards, the NiO-WO_3_ catalyst layer that can promote catalyst effects, such as ionization of oxygen species and spillover effect, was formed by the co-sputtering method via the RF magnetron sputtering system (ITS, Daejeon, Korea; PG600A_600W) on GNWs/Si substrate. In this process, in order to obtain the optimal composition and catalytic ability of NiO-WO_3_, we maximized mean free path (MFP), maintaining high vacuum for 10 h. As main sputtering after pre-sputtering for 5 min, the co-sputtering method was used under the following conditions: W target (99.95% purity), Ni target (99.95% purity), RF power (200 W), Ar flow rate (40 sccm), O_2_ flow rate (10 sccm), synthesis time (10 min), substrate rotation speed (1700 rpm), and working pressure (15 mTorr). Lastly, secondary GNWs were grown on the NiO-WO_3_/GNWs/Si substrate in the same conditions, except for the synthesis time (herein, synthesis time is 3 min).

### 2.2. Measurement and Analysis

The morphology of the GNWs/NiO-WO_3_/GNWs heterostructure was observed via a field emission scanning electron microscopy (FESEM, hitachi, Japan; S-4800) with 10 kV acceleration voltage and 9800 nA emission current. Raman spectroscopy (NOST, Seongnam, Korea; FEX) was used in the following conditions: excitation laser wavelength (~531 nm), excitation laser power (0.3 mW), objective lens (50x, NA = 0.5), and spectral resolution (~1.9–2.1 cm^−1^). As shown in Figure 1b, the measurement test for gas-sensing performance of the GNWs/NiO-WO_3_/GNWs heterostructure-based gas sensor was conducted using measurement equipment (PSS, Daejeon, Korea; GASENTEST II). Herein, the concentration of NO_2_ gas was adjusted by diluting it with dry air through a mass flow controller (MFC, Andover, MKS Instrument, USA). For the certain injection and obstruction of dry air and gas, the air valve was placed between gas pipes or lines, which were operated by the air compressor (TC-BL, Yongkang, China; DC 660). The NO_2_ gas produced by this gas-adjustment process was absorbed and desorbed at the sensing layer in the test chamber, thereby displaying a resistance change of sensing layer on the monitor.

## 3. Results and Discussion

**Figure 2:**
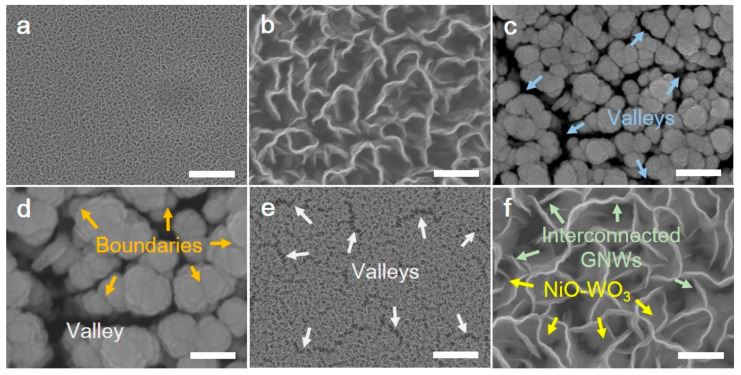
Morphology images of GNWs/NiO-WO_3_/GNWs heterostructure using FE-SEM analysis. (**a**) The morphological image of untreated GNWs (scale bar, 2 um). (**b**) The enlarged high-resolution morphology image of untreated GNWs (scale bar, 200 nm). (**c**) Co-sputtered NiO-WO_3_ structure on GNWs (scale bar, 200 nm). (**d**) The enlarged high-resolution co-sputtered NiO-WO_3_ structure with boundaries and valleys (scale bar, 100 nm). (**e**) The morphology of GNWs/NiO-WO_3_/GNWs with floating areas (scale bar, 2 um). (**f**) The enlarged high-resolution image of floating interconnected GNWs and NiO-WO_3_ (scale bar, 200 nm).

As shown in Figure 2, various morphologies of the GNWs/NiO-WO_3_/GNWs heterostructure were displayed by FE-SEM. Figure 2a,b correspond to the surface of untreated (or pure) GNWs consisting of high-density pores with a diameter of 50–200 nm (macropores). Such macropores can be a key factor to detect the gas molecules because they can induce double-sided adsorption with nanowalls in between. Considering that the diameters of nitrogen and oxygen are 0.3 nm and 0.4 nm, respectively, it is demonstrated that NO_2_ molecules can sufficiently attach at sides of nanowalls through macropores, resulting in their suitability as sensing materials. The surface images of the NiO-WO_3_-based metallic catalyst are shown in Figure 2c,d. When co-sputtering for the formation of the NiO-WO_3_ catalyst, tiny metal particles sputtered from respective targets (herein, W target and Ni target) created agglomerate composites with diameters of 120–220 nm. In general, thin films synthesized on planar substrates such as Si wafer and glass are uniform. Nevertheless, uneven surfaces with valleys between the NiO-WO_3_ agglomerations in the SEM image are due to their intensive synthesis onto the top edges of the GNWs. In addition, the surface boundaries (the orange arrows in Figure 2d) can serve as clear evidence for synthesis of NiO-WO_3_ consisting of two compositions. Secondary GNWs were then grown on NiO-WO_3_ by MPECVD, which are shown in Figure 2e,f. In contrast to the first GNWs grown on the Si substrate, the NiO-WO_3_ layer was faintly observed between the pores of the secondary GNWs with valleys. Interestingly, interconnected GNWs on valleys were observed, which may be formed from the geometric characteristic of NiO-WO_3_ (the yellow arrows and hazy section under GNWs). This suggests the growth principal of GNWs based on radical reaction from nucleation, thereby forming a nanostructure. Consequently, using PECVD and the co-sputtering system, the GNWs/NiO-WO_3_/GNWs heterostructure was ideally synthesized.

**Figure 3:**
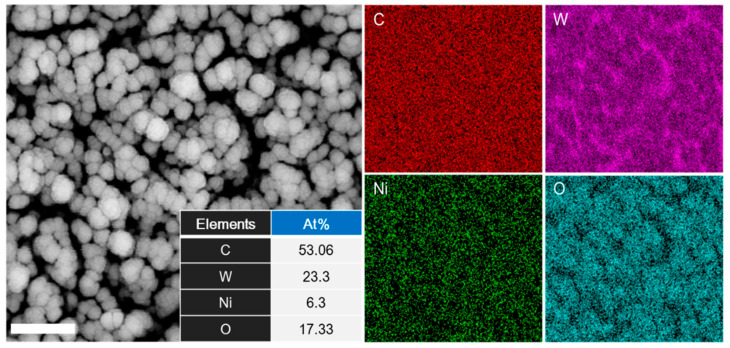
The comparison of the contents of carbon (C), tungsten (W), nickel (Ni), and oxygen (O) elements, calculated using EDS mapping (scale bar, 300 nm).

To analyze the composition ratio of the NiO-WO_3_ layer depending on the co-sputtering method, energy-dispersive X-ray spectroscopy was employed. As shown in Figure 3, mapping results of the energy-dispersive X-ray spectroscopy showed that three elements, tungsten, nickel, and oxygen, were finely distributed onto the GNWs. In the contents of the elements, the high purity of carbon demonstrated that GNWs still existed without any damages or loss by the sputtering process. In addition, when comparing the elemental contents between the tungsten and the nickel, the numerical difference is due to the type of magnetic materials. For example, as tungsten is paramagnetic, a lot of particles from the tungsten target are formed in the co-sputtering process. Whereas, in the case of the nickel, it is one of the ferromagnetic materials, which have a comparatively higher binding energy than tungsten. This implies that when nickel is sputtered in the chamber, a magnetic field formed by the magnetic characteristic of nickel may affect the plasma state, suggesting that the amount of particles reaching the substrate is inevitably low. Consequently, a catalyst layer mixed with two core catalyst materials for functionalization of the GNWs was effectively synthesized via the reactive co-sputtering system, including the three elements, oxygen, nickel, and tungsten.

**Figure 4:**
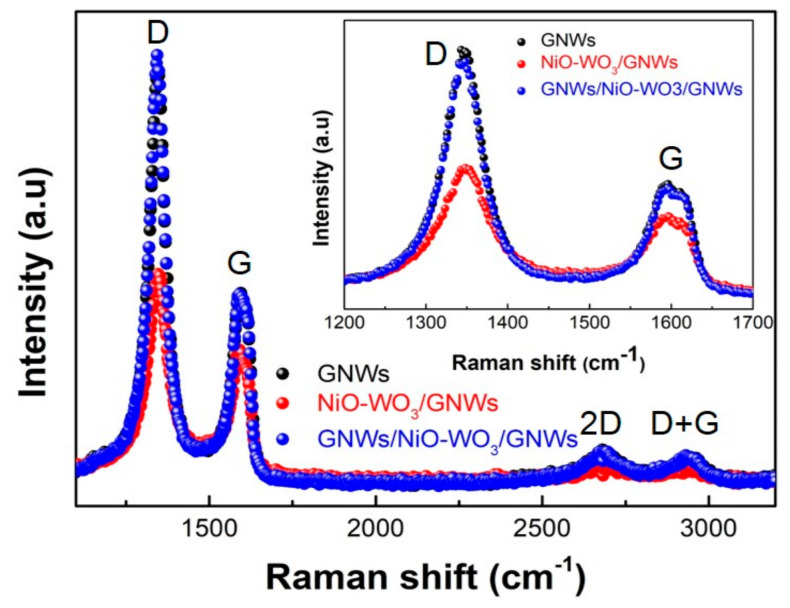
Raman spectra of the GNWs/NiO-WO_3_/GNWs heterostructure with enlarged D and G bands (inset).

Figure 4 shows the Raman spectra of the GNWs (black scatters), the NiO-WO_3_/GNWs (red scatters), and the GNWs/NiO-WO_3_/GNWs (blue scatters). As crucial peaks, D (ca. 1350 cm^−1^), G (ca. 1580 cm^−1^), and 2D band (ca. 2700 cm^−1^) occurred in all samples. Typically, the high proportion of sp^3^ hybridization is significantly higher than the sp^2^ hybridization that was seen in the GNWs. In addition, the tops of the GNWs are similar to the graphene edges in their perpendicular structure. Hence, GNWs have a high D band and a low G band, unlike graphene and carbon nanotubes. The intensity ratio of the GNWs, NiO-WO_3_/GNWs, and GNWs/NiO-WO_3_/GNWs showed numerical variation. In this case, the I_D_/I_G_ ratios of 2.14 (GNWs), 1.55 (NiO-WO_3_/GNWs), and 2.24 (GNWs/NiO-WO_3_/GNWs) were confirmed, where a notable change is a lowered value from 2.14 to 1.55. This decrease in intensity suggests that the NiO-WO_3_/GNWs was formed as composites. Nevertheless, the I_2D_/I_G_ ratio was retained at less than 1, which means that the GNWs consist of multiple graphene layers.

**Figure 5:**
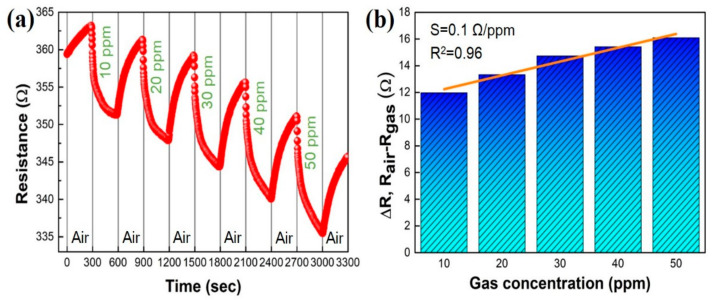
NO_2_ gas-sensing performance of GNWs/NiO-WO_3_/GNWs heterostructure-based gas sensor. (**a**) Resistance response depending on NO_2_ concentration. (**b**) Sensitivity and coefficient of determination (R^2^) by ΔR-versus-gas concentration.

Figure 5 shows the NO_2_ gas-sensing performance of the GNWs/NiO-WO_3_/GNWs heterostructure. First, as shown in Figure 5a, air and NO_2_ gas were injected at intervals of 300 s, thereby confirming the changing resistance value under a range of 10 to 50 ppm. The initial resistance of the GNWs/NiO-WO_3_/GNWs heterostructure-based gas sensor before gas injection was 363.21 Ω. As the NO_2_ concentration was increased, the resistance was reduced, while resistance increase occurred in air injection. This suggests that when the NO_2_ molecules are adsorbed onto the surface of the sensing layer, charge transfer was conducted depending on the amount of NO_2_ concentration, which directly caused the resistance to vary. Based on this result, change of resistance depending on NO_2_ concentration is shown in Figure 5b, illustrating the lowest and highest values by disparity of NO_2_ concentration 11.97 (10 ppm) and 16.10 Ω (50 ppm), respectively. In the field of sensors, sensitivity is defined as slope (herein, s = ΔR/gas concentration). The sensitivity and coefficient of determination obtained by ΔR-versus-gas concentration were 0.1 Ω/ppm and 0.96, respectively in this study. The coefficient of determination is a factor signifying the linear correlation between the *x*-axis and *y*-axis variables, which shows a high reliability of linearity closer to 1. Herein, the coefficient of determination was 0.96, which indicates that these findings are very reliable. Moreover, initial resistance continuously decreased with repetition of the injection-recovery process. We have hypothesized that it is probably because target gas adsorbed on the surface of the sensing materials still remain a small minority during the recovery step. Another cause may be due to the vertical nanostructure of the GNWs. The vertical nanostructure has rather deep pores, in which deeply penetrated gas molecules, by Knudsen diffusion, can be limited on total desorption. Thus, to keep a stable pattern of sensing signal and maintain initial resistance, an additional platform with ultraviolet (UV) light or high temperature has been used, which can be chosen to improve overall sensing ability in three-dimensional sensing materials [29,30].

**Figure 6:**
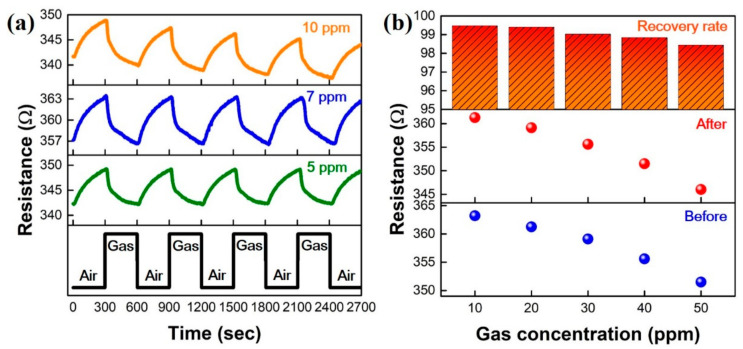
Repeatability and recovery characteristic in NO_2_ gas-sensing performance of GNWs/NiO-WO_3_/GNWs heterostructure-based gas sensor. (**a**) Repeatability at low NO_2_ concentration. (**b**) Recovery characteristic on range of NO_2_ concentration from 10 to 50 ppm.

To identify the repeatability of the GNWs/NiO-WO_3_/GNWs-based gas sensor under consistent concentration, measurements in 5, 7, and 10 ppm were conducted over several times, as shown in Figure 6a. Under conditions of alternate injection of air and gas for 300 s, outstanding repeatability was confirmed. In the repetitive test at 5 and 7 ppm, the measured average resistances were 7 and 9.16 Ω, respectively, displaying identical signal patterns. This may be because the adsorption and desorption of NO_2_ molecules are highly stable at low concentration and room temperature. However, the repeatability test at 10 ppm showed an interesting phenomenon, drawing a pattern that does not fully recover at room temperature. This phenomenon is similar to the response characteristic according to 10–50 ppm observed previously. Herein, we can infer that NO_2_ molecules adsorbed from more than 10 ppm were not thoroughly desorbed in the recovery interval and at room temperature, thereby demonstrating a phenomenon to lower the resistance value before gas response by successive accumulation of NO_2_ molecules. Figure 6b shows the recovery characteristic according to a concentration from 10 up to 50 ppm. The numerical gaps between before and after the gas response were 1.94, 2.15, 3.51, 4.11, and 5.49 Ω, respectively. The recovery rates are defined as the ratio after exposure/before exposure to gas in this section, and at each concentration they were close to 100%, with a minimum of 98.4% and a maximum of 99.47%. As mentioned at 10 ppm in Figure 6a, it can be seen that the recovery rates decrease as the concentration increases. This may be because the molecules adsorbed at more than 10 ppm still remained at a low amount at the recovery step, which is similar to the symptoms confirmed in the repeatability test.

**Figure 7:**
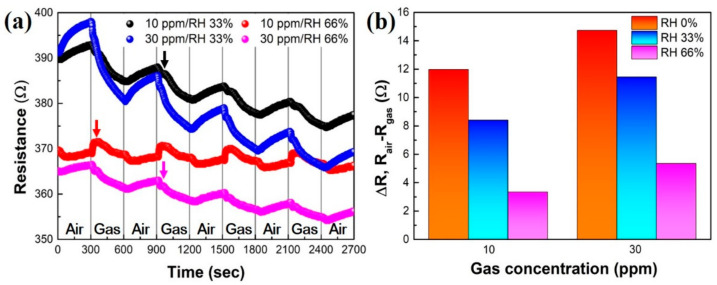
Humidity effect for GNWs/NiO-WO_3_/GNWs heterostructure-based gas sensor. (**a**) Response depends on humidity in NO_2_ gas. (**b**) Tendency of resistance change based on ΔR-versus-gas concentration.

On measurement of gas-sensing performance, since the relative humidity (RH) is a very important factor in room temperature, a humidity test was evaluated at RH 33% and RH 66% (as shown in Figure 7). Figure 7a shows the tendency that the resistance was inversely proportionate to the humidity. This may be because NO_2_ gas is diluted by humidity. Moreover, when there was no significant difference between the gas concentration and humidity, an ordinary response curve was showed, whereas in the case of a large amount of humidity, a response delay (black, red, and magenta arrows) was recorded at the initial stage, with a low response signal. Figure 7b exhibits the resistance-change-versus-gas concentration (10 and 30 ppm) at each humidity from 0 to 66%. Consequently, the GNWs/NiO-WO_3_/GNWs heterostructure-based gas sensor was greatly affected by the humidity, and it denotes that introduction of an ultraviolet irradiation system leading to enhanced stability in humidity during exposure to suitable UV wavelengths may be required to overcome the effects of humidity [31].

## 4. Conclusions

In this study, the GNWs/NiO-WO_3_/GNWs heterostructure-based NO_2_ gas sensor was reported. In morphological results of GNWs/NiO-WO_3_/GNWs heterostructure-based NO_2_ gas sensor, diverse valleys were discovered. Notably, secondary GNWs with valleys grown on the valleys of NiO-WO_3_/GNWs can offer more activation sites of NO_2_ gas molecules. From Raman spectra, the intensity ratio, by changing the D and G bands in each sample, definitely suggested the formation of the heterostructure. The GNWs/NiO-WO_3_/GNWs heterostructure-based NO_2_ gas sensor showed good performance in low concentration. The decreased tendency in resistance change occurred with an increase in gas concentration from 10 to 50 ppm. The sensitivity and R^2^ were 0.1 Ω/ppm and 0.96, respectively. The repeatability at 5–7 ppm showed great stability according to gas and air injection. We also confirmed high recovery rates of over 98%. As a result, the GNWs/NiO-WO_3_/GNWs heterostructure-based NO_2_ gas sensor has the following merits: (i) it is specialized in good stability at low gas concentration (under 7 ppm); and (ii) it exhibited hardly any difference before and after gas exposure. However, issues such as the effect of relative humidity and variation of initial resistance must surely be improved. Through these results, we found that the potential of GNWs enable us to utilize them for detection of hazardous gases such as SOx, NOx, and VOCs with necessity of further research on humidity dependence, enhanced-sensing signal, and low limit of detection.

## Data Availability

Not applicable.
